# Effects of Ramipril and Telmisartan on Plasma Concentrations of Low Molecular Weight and Protein Thiols and Carotid Intima Media Thickness in Patients with Chronic Kidney Disease

**DOI:** 10.1155/2016/1821596

**Published:** 2016-11-02

**Authors:** Angelo Zinellu, Salvatore Sotgia, Arduino A. Mangoni, Elisabetta Sotgiu, Sara Ena, Dionigia Arru, Stefano Assaretti, Angela Baralla, Andrea E. Satta, Ciriaco Carru

**Affiliations:** ^1^Department of Biomedical Sciences, University of Sassari, 07100 Sassari, Italy; ^2^Department of Clinical Pharmacology, School of Medicine, Flinders University, Bedford Park, SA 5042, Australia; ^3^Department of Surgical, Microsurgical and Medical Sciences, University of Sassari, 07100 Sassari, Italy

## Abstract

Hypertension, a common feature in chronic kidney disease (CKD), is an independent risk factor for CKD progression and cardiovascular disease. Although inhibitors of the renin-angiotensin system (RAS) exert salutary effects on blood pressure control and proteinuria in CKD patients, their activity towards traditional and novel oxidative markers is largely unknown. We studied the effects of 6-month treatment with telmisartan versus a combination of telmisartan and ramipril on plasma concentrations of low molecular mass (LMW, including homocysteine and cysteine) and protein thiols (PSH) plasma concentration and their relationships with carotid intima media thickness (IMT), in 24 hypertensive CKD patients (age 60 ± 12 years, 8 females and 16 males). Pretreatment PSH concentrations were independently associated with IMT (*r* = −0.42, *p* = 0.039). Neither treatment affected plasma LMW thiols, in both reduced and total form. By contrast, both treatments increased PSH plasma concentrations and reduced IMT, although significant differences were only observed in the combined treatment group. Our results suggest that the beneficial effects of combined RAS inhibitor treatment on IMT in hypertensive CKD patients may be mediated by a reduction of oxidative stress markers, particularly PSH.

## 1. Introduction

Hypertension is present in more than 80% of patients with chronic kidney disease (CKD) and contributes to progression towards end stage renal disease (ESRD) as well as an increased risk of cardiovascular events such as myocardial infarction and stroke [1], leading to non-CKD-related causes of death in this group. Moreover, there is good evidence that the presence of hypertension in CKD is associated with structural alterations of the arterial wall, in particular an increased thickness of the intima media layers [2]. Carotid IMT has been shown to independently predict adverse clinical outcomes in CKD [3]. Although a prolonged increase in blood pressure might be directly responsible for the development and the progression of IMT [4], other factors are likely to be involved. For example, a recent study has identified the role of oxidative stress in the development of carotid IMT in young CKD [5]. Evidence from a large number of clinical trials has clearly demonstrated that adequate blood pressure control is key to preventing adverse outcomes in CKD [6]. Inhibitors of the renin-angiotensin system (RAS), such as angiotensin converting enzyme inhibitors (ACEIs) and angiotensin II receptor blockers (ARBs), have been found to slow the progression of CKD to ESRD [7, 8]. Recent studies have also compared the effects of different RAS inhibitors. For example, in the ONTARGET study the ARB telmisartan had similar cardiovascular and renal protective effects to the ACEI ramipril in patients at high cardiovascular risk but was better tolerated [9]. Despite the established beneficial effects of RAS inhibitors in reducing proteinuria and in slowing the rate of CKD progression, direct mechanistic comparisons between ARBs and ACEIs, including possible effects on carotid IMT, have been poorly addressed. Although recent reports support the hypothesis that RAS inhibition by either ramipril or telmisartan suppresses inflammatory and lipid peroxidation markers in nonhypertensive diabetic patients [10], there is little information about other important markers of oxidation and cardiovascular risk such as LMW thiols, for example, homocysteine (Hcy) or cysteine (Cys), and their redox status modification during therapy. The highly reactive sulphur-containing amino acid Hcy has long been shown to exert detrimental effects on vascular homeostasis by inhibiting nitric oxide synthesis and promoting oxidative stress and inflammation [11]. Several studies have shown that higher plasma/serum homocysteine concentrations independently predict adverse cardiovascular outcomes [12]. Similar results have also been reported for cysteine [13, 14]. In CKD patients both Hcy and Cys plasma concentrations are commonly increased thus contributing, at least in part, to the high cardiovascular risk in this group [15]. Plasma LMW thiols also include cysteinylglycine (CysGly), glutathione (GSH), and glutamylcysteine (Glucys). These LMW thiols interact* via* redox disulfide exchange reactions and reduced, free-oxidized, and protein-bound forms of these species form a dynamic system referred to as thiols redox status that also comprises the free –SH groups of proteins (PSH) [16]. Since CKD patients are normally characterized by an increased oxidative stress and RAS inhibitors are reported to be able to reduce oxidative markers in diabetic mice [17], evaluation of total –SH groups in plasma may provide a more comprehensive assessment of the possible beneficial effects of RAS inhibition on oxidative status as well as carotid IMT. Thus, the aim of our study was to assess and compare the effects of 6-month treatment with telmisartan (80 mg day), a relatively new ARB with better tolerability, and relatively long duration of action in virtue of its longer half-life, versus a combination of telmisartan and ramipril (40/5 mg day) on LMW and PSH plasma concentrations and carotid IMT in patients with CKD. The objective was to evaluate whether the salutary effects of telmisartan on the outcomes of interest were augmented when combined with ramipril.

## 2. Methods

### 2.1. Subjects

Recruitment was performed as previously described [15]. In brief, 24 CKD hypertensive patients (age 60 ± 12 years, 8 females and 16 males) were identified at the Istituto di Patologia Medica, Azienda Ospedaliero Universitaria, with the following inclusion criteria: age > 18 years, blood pressure ≥ 140/90 mmHg, plasma LDL-cholesterol concentrations > 100 mg/dL (without concomitant hypolipidemic drugs), and presence of proteinuric CKD defined as creatinine clearance > 20 mL/min/1.73 m^2^ combined with urinary protein excretion rate > 0.3 g/24 h, without evidence of urinary tract infection or overt heart failure (New York Heart Association class III or more). Patients were of CKD stage 3 or 4, not receiving dialysis. None of the selected patients took any antihypertensive medications before the enrollment. Exclusion criteria were previous or concomitant treatment with steroids, anti-inflammatory and immunosuppressive agents, vitamin B6, vitamin B12, folate, or statin and evidence or clinical suspicion of renovascular disease, obstructive uropathy, type 1 diabetes, and vasculitis.

Enrolled patients were randomized to receive 6-month treatment with telmisartan (80 mg/day) versus a combination of telmisartan and ramipril (40/5 mg/day). Patients were assessed at baseline and at the end of treatment. Informed consent was obtained from each patient. The study was approved by our institution ethics committee and complied with the principles of the Helsinki Declaration.

### 2.2. Biochemical Analysis

Reduced and total LMW thiols were determined by capillary electrophoresis LIF detection as previously described [16, 18]. The LMW thiol redox status was calculated by measuring the ratio of the sum of reduced (r) and total (t) forms of thiols as follows: (rCysGly + rCys + rHcy + rGSH + rGluCys)/(tCysGly + tCys + tHcy + tGSH + tGluCys).

Plasma protein SH (PSH) determination was performed by spectrophotometry with 5,5′-dithiobis-2-nitrobenzoic acid (DTNB) as titrating agent by measuring the absorbance of conjugate at 405 nm [19]. Concentration in samples was determined from a GSH standard curve. Proteins –SH concentrations were normalized versus protein plasma quantity measured by Lowry's method.

### 2.3. Blood Pressure Measurement

Blood pressure (BP) was measured at the Istituto di Patologia Medica, Azienda Ospedaliero Universitaria, by a trained research assistant using a calibrated and validated digital sphygmomanometer. BP measurements were taken with the patient in a seated position with the arm supported at heart level, after a 5 min rest and after abstaining from food, beverages containing caffeine, and smoking for at least 2 h prior to BP measurement. BP was recorded as three serial measurements at intervals of 30 sec on both arms. The mean of the six BP readings was used in the analysis. If a BP reading deviated by more than 10 mmHg from the average reading, the BP measurement on that arm was repeated.

### 2.4. Carotid IMT Measurement

The carotid artery was scanned by two trained and certified sonographers blinded to clinical information and treatment. All study subjects were examined in the supine position with the head tilted backward. Dedicated QIMT-Esaote software was used to measure the left and right IMT of the posterior wall of the common carotid, 1 cm distal to the bulb bifurcation, by ultrasound 2D exam. The IMT assessment was performed according to the recommendations of the Italian Society of Vascular Diagnostics [20] and the standardized criteria described by Mannheim Carotid Intima Media Thickness Consensus to ensure full reliability [21]. The manual image acquisition of carotid vessels was obtained before the automatic measurement of IMT using QIMT (Quality Intima Media Thickness) software, integrated into the instrument (Esaote MyLab 30 gold), and QIMT software, using the RF (Radio Frequency Data Processing). Two readings from both common carotid arteries were averaged to calculate the mean IMT. A thickness of >0.8 mm indicated increased IMT.

### 2.5. Statistical Analysis

All results are expressed as mean values (mean ± SD) or median values (median and interquartile range). The variables distribution was assessed by the Shapiro-Wilk test. Homogeneity of variance was checked by *F*-test and differences between groups were compared by nonparametric Mann-Whitney *U* test or by parametric independent *t*-test, with or without Welch's correction for unequal variances, as appropriate. Nonnormally distributed variables were log⁡10-transformed prior assessment with parametric tests, and the normal distribution of the residuals was checked to assess the goodness of fit of the transformations. Correlation analysis between variables was performed by Pearson's correlation or Spearman's correlation. Multiple linear regression analysis was used to assess the association between different variables and IMT at baseline. The effect of treatment was evaluated by ANOVA.

Statistical analyses were performed using MedCalc for Windows, 12.5 64-bit version (MedCalc Software, Ostend, Belgium), and SPSS for Windows, 14.0 32-bit version (IBM Corporation, Armonk, NY, USA).

## 3. Results

**Figure 1:**
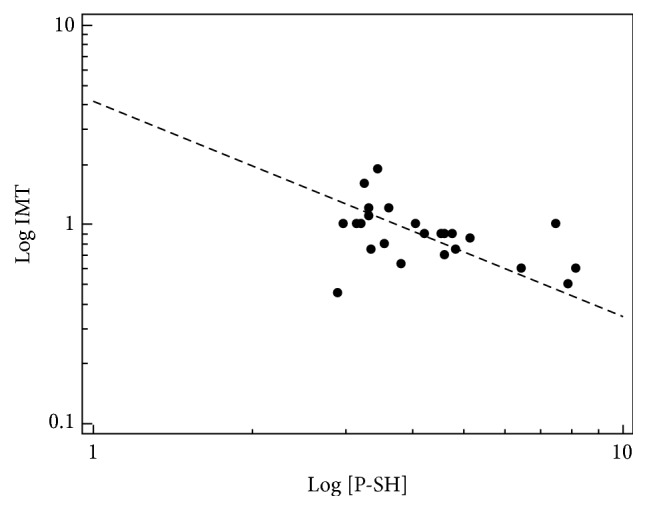
Correlation between carotid IMT and PSH in CKD patients at baseline.

Clinical characteristics of CKD patients at baseline are described in Table 1. After randomization, no significant differences were found among the two treatment groups in the clinical characteristics (Table 1). As expected, more than 60% of CKD subjects were hyperhomocysteinemic (Hcy >15 *μ*mol/L) versus 10% normally found in healthy population. As reported in Figure 1, baseline IMT was inversely related to PSH concentrations (*r* = −0.42, *p* = 0.039). In multiple linear regression analysis, with IMT as dependent variable and age, gender, GFR, creatinine, PSH, SBP, and LMW red/ox ratio as independent variables, only PSH (*β* = −0.51, *p* = 0.027) was independently associated with baseline IMT.

As reported in Table 2, a reduction in blood pressure was observed in both groups; however it was statistically significant only in the telmisartan/ramipril group (*p* < 0.05). Creatinine and proteinuria showed a nonsignificant trend toward improvement in both groups after 6-month treatment. LMW thiols concentrations were unaffected by drug treatment both in the reduced and in the total form. In particular no differences were found in the cardiovascular risk factors homocysteine and cysteine plasma concentrations. As a result, the LMW red/ox ratio remained essentially unchanged at the end of treatment (Table 3).

**Figure 2:**
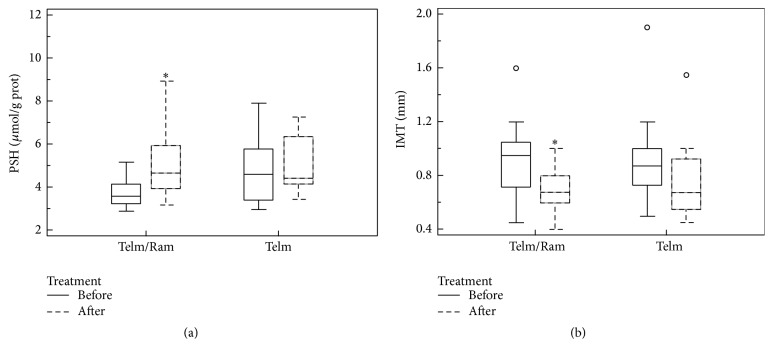
Comparison between PSH levels (a) and IMT values (b) at baseline and after six months of therapy. ^*∗*^ *p* < 0.05.

By contrast, as shown in Figure 2(a), PSH plasma concentrations significantly increased in the telmisartan/ramipril group (median: 3.59 *μ*mol/g prot (3.31–4.80 *μ*mol/g prot) at baseline versus 4.66 *μ*mol/g prot (3.94–5.96 *μ*mol/g prot) after treatment, *p* = 0.015) whereas they remained essentially unchanged in the telmisartan group (median: 4.61 *μ*mol/g prot (3.39–5.80 *μ*mol/g prot) at baseline versus 4.43 *μ*mol/g prot (4.16–7.27 *μ*mol/g prot) after treatment). Carotid IMT decreased in both groups (Figure 2(b)). However, the reduction was statistically significant only in the telmisartan/ramipril group [telmisartan/ramipril: median 0.95 mm (0.72–1.05 mm) at baseline versus 0.68 mm (0.60–0.80 mm) after treatment, *p* = 0.027; telmisartan: median 0.88 mm (0.73–1.00 mm) at baseline versus 0.68 mm (0.55–0.93) after treatment].

## 4. Discussion

A number of trials and meta-analyses have demonstrated that combined treatment with ACEIs and ARBs exerts greater effects on blood pressure lowering and proteinuria than agent alone [22, 23]. However, combination therapy may also result in a significant increased incidence of adverse effects, as demonstrated in the ONTARGET trial of 25,620 patients with preexisting vascular disease or diabetes. ONTARGET was designed to evaluate the effects of ramipril, telmisartan, or their combination on cardiovascular and renal endpoints during approximately 4.5 years of follow-up [24]. In this study, a small but significant increase in the incidence of ESRD, hyperkalemia, and hypotension was observed with the combination therapy [24]. Moreover, the combination treatment was not associated with better clinical outcomes when compared with each treatment alone [25].

In view of the ongoing debate on the efficacy and safety of combined RAS inhibitors treatment and the uncertainties regarding the putative mechanisms responsible for the beneficial effects of these drug class, we assessed and compared the effects of telmisartan administration alone (80 mg day) versus a combination of telmisartan/ramipril (40/5 mg day) on plasma LMW and protein thiols concentrations and carotid IMT in CKD patients. Our data confirm previous observations that dual RAS blockade has greater blood pressure effects if compared with telmisartan alone [24]. For the first time, we comprehensively evaluated the effect of RAS inhibitor therapy on low molecular mass thiols Hcy and Cys (two well known risk factors for cardiovascular disease) both in the total and in the reduced form. As expected, CKD was associated with relatively high plasma concentrations of both total Cys and Hcy. RAS treatment did not exert any significant effect on plasma low molecular mass thiols (for both reduced and total forms) so that LMW thiols redox status remains unchanged after therapy. Conversely, combined treatment significantly increased PSH concentrations. PSH values provide a comprehensive measure of total protein sulfhydryl groups in plasma. The most abundant reduced -SH group in plasma is that of human serum albumin (HSA), given its high concentrations. The single free cysteinyl thiol of HSA, Cys^34^, accounts for ~80% of reduced thiols in human plasma and is an important scavenger of reactive oxygen and nitrogen species in the vascular compartment, thus a quantitatively important redox buffer of blood [26]. Therefore, in the presence of reactive oxygen species the PSH concentrations decrease, whereas an increment in PSH concentrations indicates oxidative stress reduction. Our data on PSH are in agreement with previous reports showing that ramipril effectively decreased oxidative stress indices [17].

Notably, the salutary effects of RAS combination treatment were paralleled by a concomitant reduction in carotid IMT. As baseline PSH was the only parameter independently associated with IMT, we speculate that the changes in IMT during combination treatment may be mediated, at least in part, by the reduction of oxidative stress. This hypothesis is supported by previous studies showing an inverse association between IMT and plasma total antioxidant capacity [27].

There are some limitations in this study that deserve mention. The data were obtained from a relatively small study population and did not include a placebo group. Therefore, our results require confirmation in larger study cohorts. The treatment duration, 6 months, is relatively short if compared to previous studies, reporting follow-ups between 2 and 4 years. Finally, the applicability of our results to populations other than CKD remains to be established, in view of the peculiar proinflammatory and oxidative stress state of these patients.

## 5. Conclusion

We found that 6-month RAS inhibitor treatment in CKD with either telmisartan alone or in combination with ramipril did not significantly affect LMW plasma thiols in both the reduced and the total form, particularly Hcy and Cys. By contrast, a significant reduction in carotid IMT and a concomitant increase in PSH plasma concentrations were observed in the combination treatment group. These results provide novel information on the potential mechanistic effects of RAS inhibition on carotid IMT, a key marker of vascular remodeling and independent predictor of adverse events in hypertension and CKD. Further studies in larger cohorts with longer follow-ups and other established surrogate risk markers are required to confirm the hypothesis that the IMT improvement with RAS inhibition is mediated by oxidative stress decrease.

## Figures and Tables

**Table 1:** Demographic and clinical characteristics of CKD randomized groups.

	CKD (*n* = 24)	Group 1 (*n* = 12) *Telmisartan/ramipril* *40/5 mg/day*	Group 2 (*n* = 12) *Telmisartan* *80 mg/day*
	Mean ± SD or median (IQR)	Mean ± SD or median (IQR)	Mean ± SD or median (IQR)
Sex, F/M (% F)	8/16 (33%)	4/8 (33%)	4/8 (33%)
Age, years	60 ± 12	62 ± 12	58 ± 13
Systolic BP, mmHg	133 ± 13	132 ± 13	133 ± 14
Diastolic BP, mmHg	80 ± 9	82 ± 8	77 ± 9
Creatinine, mg/dL	1.6 (1.1–2.1)	1.6 (1.0–2.3)	1.7 (1.3–2.0)
GFR, mL/min per 1.73 m^2^	48 (32–66)	48 (22–71)	48 (34–66)
Proteinuria, g/24 h	0.95 (0.30–2.35)	0.52 (0.28–2.49)	1.28 (0.36–2.35)
IMT, mm	0.90 (0.73–1.00)	0.95 (0.72–1.05)	0.88 (0.73–1.00)
*Total LMW thiols*			
t-CysGly, *µ*mol/L	30.2 ± 8.6	31.6 ± 8.8	28.8 ± 8.4
t-Hcy, *µ*mol/L	17.0 (12.1–24.4)	16.3 (12.1–26.3)	19.2 (11.8–23.7)
t-Cys, *µ*mol/L	381 ± 98	391 ± 89	370 ± 109
t-GSH, *µ*mol/L	7.5 ± 2.6	7.4 ± 2.1	7.6 ± 3.2
t-GluCys, *µ*mol/L	5.8 (3.9–6.5)	5.7 (4.5–6.0)	5.4 (3.5–7.6)
Sum of total LMW thiols, *µ*mol/L	444 ± 112	458 ± 106	432 ± 122
*Reduced LMW thiols*			
r-Cys-Gly, *µ*mol/L	6.2 ± 2.2	5.8 ± 1.6	6.6 ± 2.6
r-Hcy, *µ*mol/L	0.24 (0.15–0.37)	0.20 (0.13–0.37)	0.29 (0.18–0.37)
r-Cys, *µ*mol/L	21.6 ± 4.1	20.9 ± 4.7	21.9 ± 3.5
r-GSH, *µ*mol/L	1.09 (0.78–1.46)	1.01 (0.74–1.31)	1.11 (0.85–1.67)
r-Glu-Cys, *µ*mol/L	0.36 (0.26–0.46)	0.35 (0.22–0.58)	0.37 (0.27–0.44)
Sum of reduced LMW thiols, *µ*mol/L	29.4 ± 5.9	28.4 ± 5.9	30.5 ± 6.0
*Thiols redox status*			
LMW redox, %	0.072 ± 0.027	0.066 ± 0.023	0.078 ± 0.030
PSH, *µ*mol/g prot	3.95 (3.31–4.80)	3.59 (3.23–4.15)	4.61 (3.39–5.80)

**Table 2:** Drug effects on blood pressure and renal function markers.

	Group 1 (*n* = 12)			Group 2 (*n* = 12)		
	*Baseline*	*Telmisartan/ramipril* *40/5 mg/day*	*p* value	*Baseline*	*Telmisartan* *80 mg/day*	*p* value
	Mean ± SD or median (IQR)	Mean ± SD or median (IQR)		Mean ± SD or median (IQR)	Mean ± SD or median (IQR)	
Systolic BP, mmHg	132 ± 13	122 ± 10	*0.04*	133 ± 14	123 ± 15	*0.11*
Diastolic BP, mmHg	82 ± 8	75 ± 5	*0.02*	77 ± 9	76 ± 10	*0.80*
Creatinine, mg/dL	1.6 (1.0–2.3)	1.5 (1.1–2.2)	*0.57*	1.7 (1.3–2.0)	1.5 (1.3–2.0)	*0.85*
GFR, mL/min per 1.73 m^2^	48 (22–71)	47 (23–61)	*0.79*	48 (34–66)	55 (36–71)	*0.79*
Proteinuria, g/24 h	0.52 (0.28–2.49)	0.49 (0.22–1.15)	*0.07*	1.28 (0.36–2.35)	0.67 (0.44–1.68)	*0.08*

**Table 3:** Drug effects on LMW thiols.

	Group 1 (*n* = 12)			Group 2 (*n* = 12)		
	*Baseline*	*Telmisartan/ramipril* *40/5 mg/day*	*p value*	*Baseline*	*Telmisartan* *80 mg/day*	*p value*
	Mean ± SD or median (IQR)	Mean ± SD or median (IQR)		Mean ± SD or median (IQR)	Mean ± SD or median (IQR)	
*Total LMW thiols*						
t-CysGly, *µ*mol/L	31.6 ± 8.8	31.1 ± 8.5	*0.89*	28.8 ± 8.4	25.6 ± 7.9	*0.35*
t-Hcy, *µ*mol/L	16.3 (12.1–26.3)	17.3 (10.3–27.6)	*0.90*	19.2 (11.8–23.7)	14.4 (11.2–19.2)	*0.27*
t-Cys, *µ*mol/L	391 ± 89	404 ± 122	*0.77*	370 ± 109	341 ± 94	*0.49*
t-GSH, *µ*mol/L	7.4 ± 2.1	7.7 ± 1.8	*0.71*	7.6 ± 3.2	7.6 ± 4.6	*1.00*
t-GluCys, *µ*mol/L	5.7 (4.5–6.0)	5.7 (4.4–9.7)	*0.38*	5.4 (3.5–7.6)	4.4 (3.2–7.2)	*0.95*
Sum of total LMW thiols, *µ*mol/L	458 ± 106	472 ± 144	*0.79*	432 ± 122	400 ± 112	*0.51*
*Reduced LMW thiols*						
r-CysGly, *µ*mol/L	5.8 ± 1.6	5.5 ± 1.6	*0.65*	6.6 ± 2.6	6.4 ± 1.7	*0.83*
r-Hcy, *µ*mol/L	0.20 (0.13–0.37)	0.17 (0.12–0.36)	*0.57*	0.29 (0.18–0.37)	0.23 (0.17–0.29)	*0.42*
r-Cys, *µ*mol/L	20.9 ± 4.7	19.7 ± 4.7	*0.54*	21.9 ± 3.5	21.0 ± 4.4	*0.58*
r-GSH, *µ*mol/L	1.01 (0.74–1.31)	0.94 (0.47–1.37)	*0.91*	1.11 (0.85–1.67)	1.10 (0.71–1.60)	*0.34*
r-GluCys, *µ*mol/L	0.35 (0.22–0.58)	0.41 (0.31–0.47)	*0.85*	0.37 (0.27–0.44)	0.43 (0.33–0.45)	*0.97*
Sum of reduced sLMW thiols, *µ*mol/L	28.4 ± 5.9	26.1 ± 5.1	*0.32*	30.5 ± 6.0	29.3 ± 5.8	*0.62*
*Thiols redox status*						
LMW redox, %	0.066 ± 0.023	0.061 ± 0.025	*0.62*	0.078 ± 0.030	0.079 ± 0.028	*0.93*

## Abbreviations

ACEIs: Angiotensin converting enzyme inhibitors
ARBs: Angiotensin II receptor blockers
CKD: Chronic kidney disease
Cys: Cysteine
Hcy: Homocysteine
LMW: Low molecular weight
PSH: Protein thiols
RAS: Renin-angiotensin system.
